# Precision-Microfabricated Fiber-Optic Probe for Intravascular Pressure and Temperature Sensing

**DOI:** 10.1109/JSTQE.2021.3054727

**Published:** 2021-01-29

**Authors:** Radhika K. Poduval, Joanna M. Coote, Charles A. Mosse, Malcolm C. Finlay, Adrien E. Desjardins, Ioannis Papakonstantinou

**Affiliations:** 1 Department of Electronic and Electrical EngineeringUniversity College London 4919 LondonWC1E 7JEU.K.; 2 Department of Medical Physics and Biomedical EngineeringUniversity College London 4919 LondonWC1E 6BTU.K.

**Keywords:** Optical fiber sensors, biomedical signal detection, optical interferometry, blood pressure measurement, temperature measurement, microsensors, photolithography, optical waveguide

## Abstract

Small form-factor sensors are widely used in minimally invasive intravascular diagnostic procedures. Manufacturing complexities associated with miniaturizing current fiber-optic probes, particularly for multi-parameter sensing, severely constrain their adoption outside of niche fields. It is especially challenging to rapidly prototype and iterate upon sensor designs to optimize performance for medical devices. In this work, a novel technique to construct a microscale extrinsic fiber-optic sensor with a confined air cavity and sub-micron geometric resolution is presented. The confined air cavity is enclosed between a 3 μm thick pressure-sensitive distal diaphragm and a proximal temperature-sensitive plano-convex microlens segment unresponsive to changes in external pressure. Simultaneous pressure and temperature measurements are possible through optical interrogation via phase-resolved low-coherence interferometry (LCI). Upon characterization in a simulated intravascular environment, we find these sensors capable of detecting pressure changes down to 0.11 mmHg (in the range of 760 to 1060 mmHg) and temperature changes of 0.036 °C (in the range 34 to 50 °C). By virtue of these sensitivity values suited to intravascular physiological monitoring, and the scope of design flexibility enabled by the precision-fabricated photoresist microstructure, it is envisaged that this technique will enable construction of a wide range of fiber-optic sensors for guiding minimally invasive medical procedures.

## I. Introduction

Miniature minimally-invasive sensors allow for fine spatial resolution and targeted point measurements at critical sites, particularly for biomedical diagnostics [1], [2]. These include intrasurgical measurement of pressure and temperature in cardiac vasculature during cardiovascular electro-surgery, intracranial physiological monitoring during head trauma recovery, urological interventions, among others [3]–[9]. Particularly for minimally invasive surgical (MIS) procedures, electronic sensors or manometer-style catheters tend to be too large for *in vivo* use [10]. Moreover, the electromagnetic interference (EMI) sensitivity of electronic sensors and damping of pressure dynamics in manometric catheters can present challenges with intravascular applications. The risk of embolism, infection and thrombosis increases with the size of the sensor, highlighting the importance of sensor miniaturization [11].

Fiber-optic sensors present a favorable alternative for use in minimally invasive medical applications. Of particular interest is their immunity to EMI, magnetic resonance imaging (MRI) compatibility, and high mechanical flexibility [2], [12], [13]. When designed to have sub-mm lateral dimensions, these can be readily integrated within existing catheters, needles and guidewires [6].

Fiber-optic pressure and temperature sensors comprise one or more responsive optical cavities that are interferometrically interrogated to measure corresponding deflections. Such probes can be formed by affixing an optical cavity structure consisting of a deformable diaphragm extrinsic to a waveguiding optical fiber. Materials such as Si, SiO_2_, metal films, nano-diamond, ceramics, and organic polymers have been investigated in this regard [14]–[21]. However, obtaining the geometric tolerances needed to reliably attach these optical cavity elements to fiber-optic waveguides is challenging. Commercial fiber-optic interferometric sensors are typically formed by anodic bonding of a thin Si diaphragm onto a glass support structure or capillary [22] This method does not allow for freeform design or structural modification for prototyping and tends to be expensive in terms of both assembly time and capital equipment required. Inclusion of custom-designed optical elements for lensing or simultaneous multi-parameter measurements is also challenging using these methods.

3D laser lithography is of interest for precision microfabrication of fiber-optic sensors. Early work on this paradigm was done by Hill *et al.* to form an SU-8 based cylindrical extrinsic structure with a single air-filled optical cavity [23]. To our knowledge however, a fiber-optic sensor with lithographically formed freeform monolithic extrinsic sensing structure comprising a confined gas cavity and microlens is yet to be reported. A few combinatorial approaches with multiple integration steps have been investigated in this context,[19], [24]–[27] but a submicron resolution optical cavity structure capable of pressure and temperature sensing in a remote fluidic environment has been elusive.

**Fig. 1.**
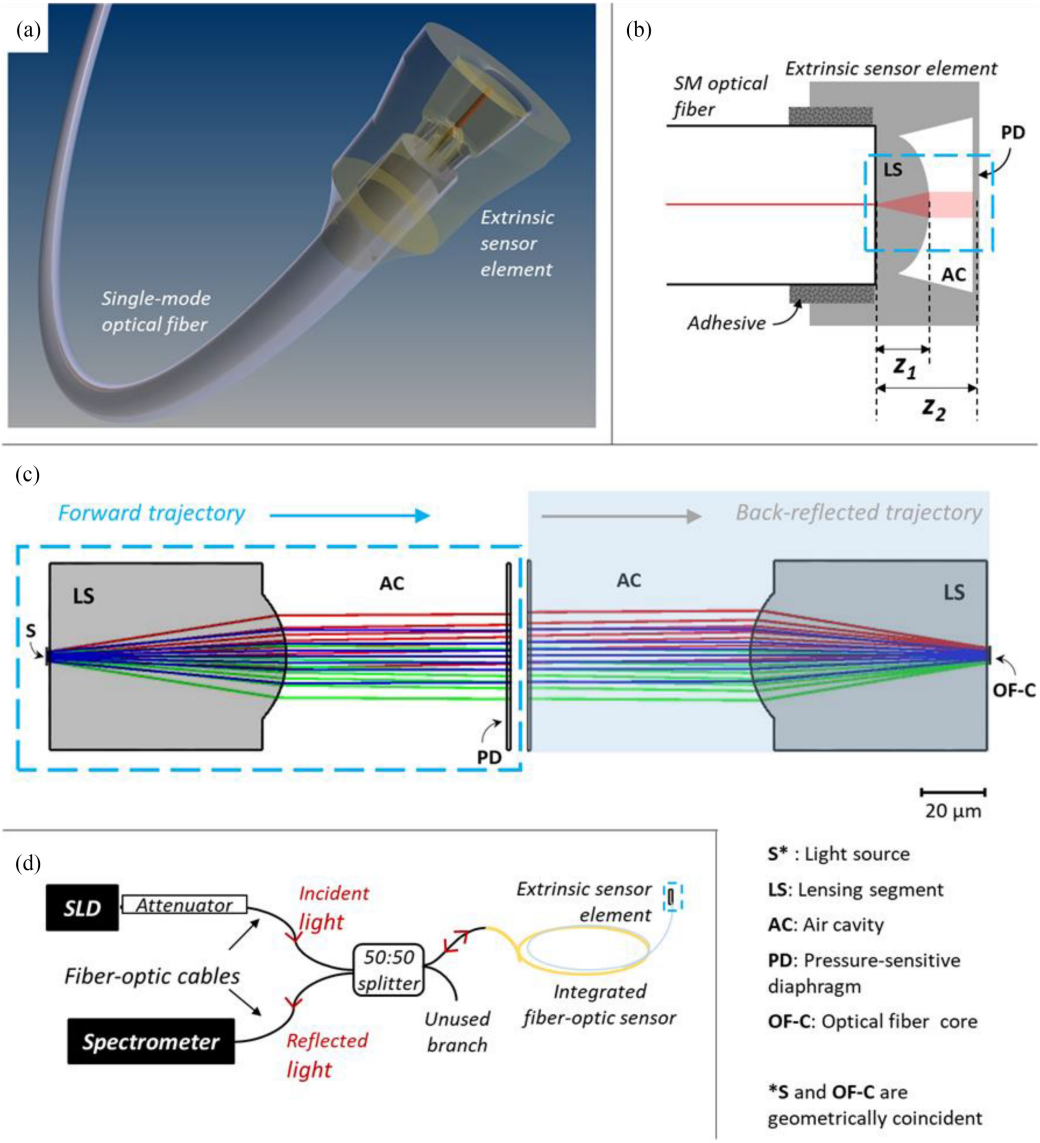
Sensing probe design. (a) CAD illustration of our multi-cavity fiber-optic probe for intravascular pressure and temperature sensing. (b) Longitudinal cross-section representation of the sensing element design of the probe with its major optical path lengths indicated as z_1_ and z_2_, (including the refractive index). The blue dashed rectangle indicates the region of significance of optical design optimization in the extrinsic sensing element. (SM: single mode, LS: lensing segment, AC: air cavity, PD: pressure-sensitive diaphragm). (c) Illustration of the ray-tracing optimization of the forward and back-reflected trajectories of the interrogation beam within the extrinsic sensor element; the forward and return beam trajectories are shown side by side; the geometry of the sensing element is flipped horizontally for the back-reflected trajectory, so that the direction of propagation of the rays is left-to-right in both images. (d) Schematic of the Low-Coherence Interferometry (LCI) interrogation setup used to interrogate the integrated fiber-optic sensor (SLD: super-luminescent diode).

In this work, we demonstrate the precision microfabrication of such a fiber-optic sensor that is simultaneously responsive to changes in both pressure and temperature within the intravascular physiological range [28]. A 3D monolithic freeform structure comprising a microscale deformable diaphragm, a confined air cavity, and an optimized lensing segment was designed for integration onto the cleaved end-face of a single-mode (SM) optical fiber, illustrated in Fig. 1(a). A key step in the fabrication process of this extrinsic sensing microstructure involved the use of two photon polymerization (TPP) printing. TPP leverages two-photon absorption (TPA) of near-infrared (NIR) radiation for additive manufacturing with sub-diffraction limit resolution within the bulk of a photosensitive material [29]–[33]. This is typically realized by high-resolution scanning of a tightly focused femtosecond laser beam across the material to induce crosslinking in the exposed regions, followed by washing out of the unprocessed material, leaving the cross-linked geometry composed of the photopolymerized resin.

TPP microfabrication of structures directly onto the cleaved end-faces of optical fibers have been reported to form lenses, gratings and micro-spring elements [34]–[37]. However, direct on-fiber TPP does not allow for confined air cavities distal to the optical fiber end-face, as open outlets with diameters exceeding 20 μm are needed to ensure complete removal of the unexposed resin during photoresist development. Additionally, the adhesion between the resist material and the fiber end-face does not ensure irreversible bonding of the TPP microstructure to the optical fiber as required for intra-surgical use. Moreover, the presence of low-reflectance deformable polymer interfaces forming low-finesse cavities presents a challenge in obtaining adequate signal to noise ratio (SNR) at low optical illumination in interferometric sensing probes.

We have overcome these challenges through a combination of freeform 3D multi-cavity sensor design, optical path raytracing, TPP and single-step microstructure bonding to an SM optical fiber. Optical path length changes of the low-finesse cavities in these sensors were interrogated by phase-resolved low-coherence interferometry (LCI). We then characterized these devices to evaluate their structural design fidelity and application potential for sensing pressure and temperature changes in the range of minimally invasive interventions in a simulated intravascular environment.

## II. Fiber-Optic Sensor Design

The extrinsic sensing element of our fiber-optic probe comprised a cylindrical cap structure with three optically reflecting interfaces along the beam path forming two distinct low-finesse optical cavities (Fig. 1(b)). Here, the pressure response is indicated by the deflection of the distal diaphragm segment at the end of this cap structure. Further, to enable sensitivity in the intravascular temperature range, the design included a 75 μm thick central polymer block segment positioned adjacent to the cleaved end-face of the SM optical fiber, to which the sensor element would be bonded. This polymer block segment is shielded from the external environment, and consequently the ambient mechanical perturbations. As a result, while being insensitive to pressure changes in the biomedical/physiological range of interest, it would be responsive to temperature changes due to the inherent thermal expansion and the thermo-optic coefficient of the monolithic polymer microstructure.

### A. Ray Tracing Design Optimization

This multi-cavity design was further modified with the aim of improving detection sensitivity and reducing the super-luminescent diode (SLD) illumination power required for LCI interrogation (see Fig. 1(d) and Materials and Methods section). Using low SLD illumination power is desirable as it reduces the likelihood of beam-induced heating effects in the extrinsic sensor element, and/or the surrounding media beyond the partially transmissive distal deformable diaphragm (for instance blood when considering the intravascular space). To enable LCI signal detection at lower illumination power, the sensor element design was modified to enhance coupling of the back-reflected beam from each cavity surface into the core of the SM optical fiber. In contrast, within the case of parallel reflecting surfaces as is the design norm for FP cavities in fiber-optic pressure sensors [19], [38]–[40], only a small fraction of the reflected interferometric signal can couple back into the SM fiber to be detected by the spectrometer. This is a result of progressive divergence during both the forward and back-reflected trajectories within the optical cavity when passing through the extrinsic sensor element.

To address this loss of signal when coupling the interrogation beam back into the optical fiber core, ray-tracing optimization of the sensor element optics design was performed. Accordingly, the central segment geometry was modified to reduce beam divergence in forward propagation, and aid coupling of the back-reflected beam into the optical fiber core on the return path. The optimized optical design, as per the raytracing simulations, is shown in Fig. 1(c), comprising a proximal circularly-symmetric plano-convex lensing segment (radius of curvature: 30 μm; thickness: 75 μm), an air cavity (length: 80 μm), and a deformable diaphragm (thickness: 3 μm) at its distal end.

**Fig. 2.**
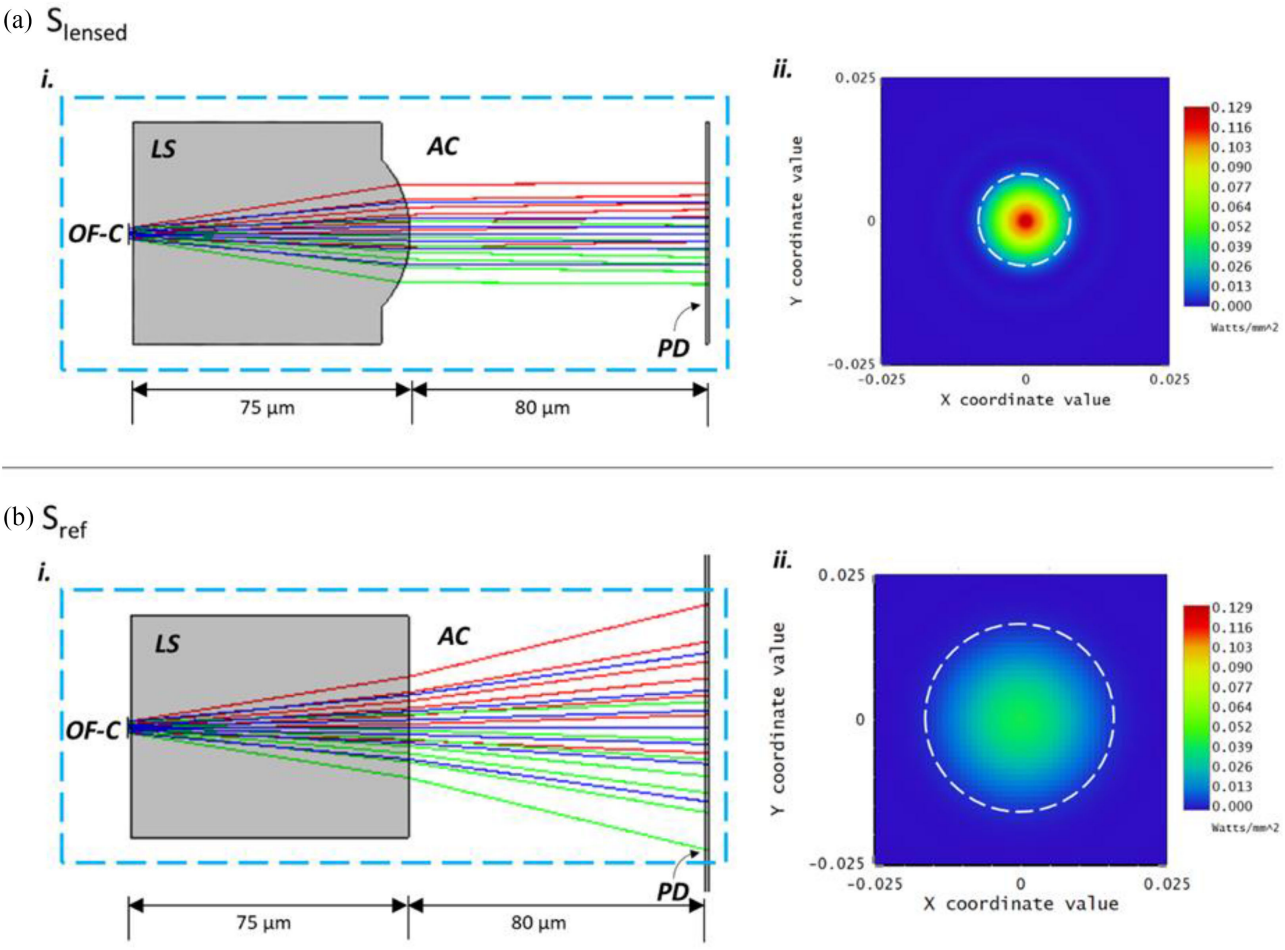
Illustration of interrogating beam spot image at the distal deformable diaphragm (PD) obtained through raytracing simulations in the (a) optimized curved distal surface S_lensed_ (radius of curvature 30 μm) vs (b) flat lensing segment S_ref_. Only the forward beam propagation is indicated here. The respective multi-cavity designs are indicated under i. and the beam spot notations simulated at the corresponding PD denoted under ii., with the measured beam spot sizes outlined by the dashed white lines (beam spot diameters 20 μm and 39 μm respectively). The X and Y coordinate values are indicated in μm units. The irradiance values were normalized to the maximum intensity in i.

Furthermore, the lensing capability of this integrated sensor (S_lensed_) was evaluated against a comparable reference sensor design (S_ref_) where the polymer block segment had a flat surface instead of a curved lensing surface, shown in Fig. 2. It was observed in raytracing simulations (details in Materials and Methods section) that the beam spot at the outer surface of the deformable diaphragm (indicated as PD) was found to diverge significantly more in S_ref_ compared to S_lensed._, yielding an almost 4-fold larger beam spot irradiance area with S_ref_.

### B. Optical Interrogation System

These fiber-optic sensors were designed for interrogation by phase-resolved low-coherence interferometry (LCI). The Michelson interferometer sensor interrogation setup used in this study is based on the miniaturized setup described by Coote *et al.* [19] This is schematically shown in Fig. 1(d) and described further in the Materials and Methods section. Light from the optical fiber is guided to the extrinsic sensing element, where each interface forms a reflective surface owing to the refractive index contrasts. Light passing through each interface is partially reflected, and returns to the waveguiding optical fiber to be analyzed by an optical spectrometer along a common path. Our sensor was designed to yield two prominent low-finesse optical cavities discernable with the interrogating LCI system (see Materials and Methods). The presence of low-finesse optical cavities causes the interference pattern observed at the spectrometer in the LCI interrogation system, and the changes in optical path length of each cavity can be monitored as variations in this interference pattern [19]. A change in the external temperature and/or pressure results in a change in the lengths of the optical cavities (termed ***Δz*_1_** and ***Δz*_2_**, considering the optical cavities highlighted in in Fig. 1(b)). Referring to the design requirement laid out for our sensor earlier in this section, variation in both external temperature and pressure is expected to induce changes in ***z*_2_**, while ***z*_2_** would only respond to changes in temperature due to thermal expansion of the TPP-printed extrinsic sensor element material.

## III. Construction of the Fiber-Optic Sensor

### A. Precision Microfabrication of the Extrinsic Sensing Element

**Fig. 3.**
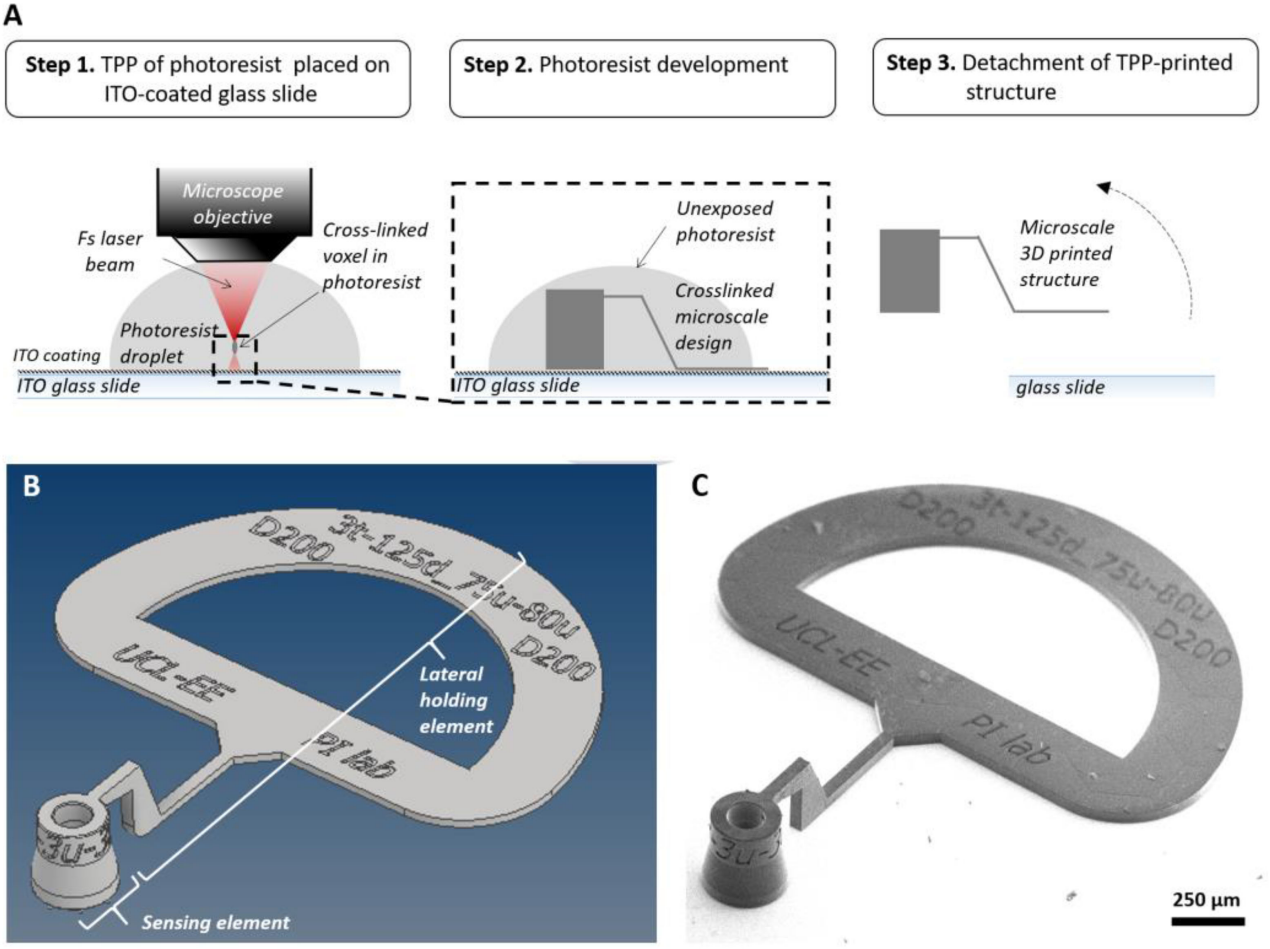
Extrinsic sensor element microfabrication. (a) Schematic illustration of the microfabrication process. TPP: two-photon polymerization, ITO-Indium Tin Oxide. (b) Isometric view of the microstructure CAD of the consolidated design for TPP. (c) SEM view of the TPP-fabricated microstructure following detachment from the glass substrate.

Using the results of the raytracing optimization, a freeform 3D computer-aided design (CAD) of the sensor element was formulated for TPP microfabrication. To simplify maneuvering the extrinsic microscale sensor element for fiber integration (indicated in Fig. 1(a)) following fabrication, an additional lateral holding element was added to the design (Fig. 3(b)).

This CAD design formed the basis of defining the femtosecond laser beam trajectory for TPP microfabrication. TPP processing parameters were modified in order to achieve smooth surface topography, in addition to reducing the X-Y and Z scanning tolerances to below 0.3 μm each. Microstructure fabrication involved laser illumination to induce TPP within a proprietary IP-S photoresist droplet (Nanoscribe) on an ITO-coated glass substrate. Photoresist development, i.e., washout of unilluminated resist, was performed by immersing in propylene-glycol-monomethyl-ether-acetate (PGMEA) (further details in Materials and Methods). Thereafter, these structures were safely detached from the substrate for integration to the 125 μm diameter SM optical fiber. A summary of these fabrication steps is shown in Fig. 3(a). A complete CAD view is shown in Fig. 3(b), while Fig. 3(c) is an SEM image of the final microfabricated structure following detachment from the ITO-glass substrate, indicating excellent geometric correspondence between the CAD design and the fabricated sensor element.

**Fig. 4.**
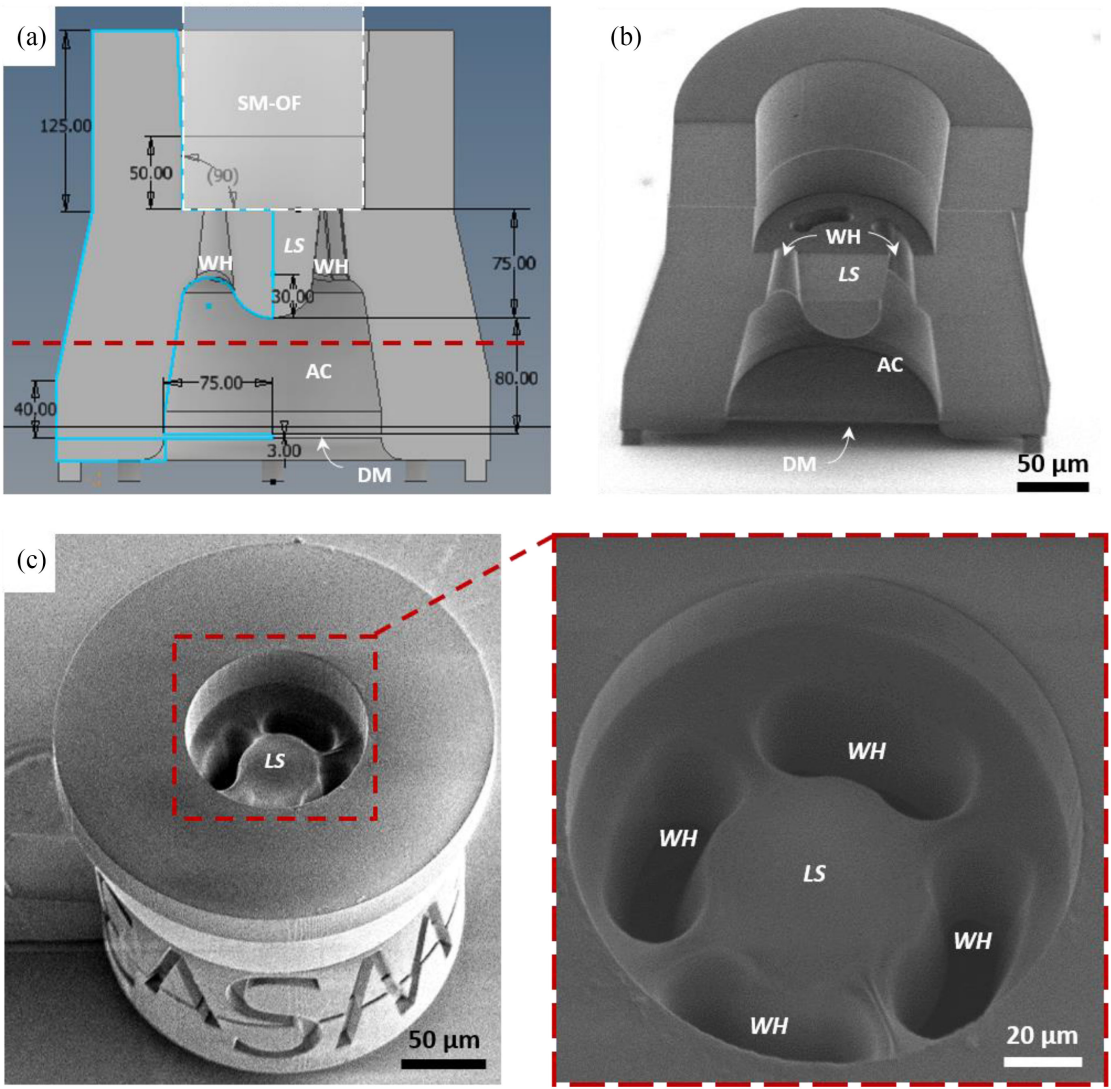
Optimized CAD geometry and cross-sectional SEM of the extrinsic sensing element. (a) Longitudinal cross-section CAD view of the optimized sensor element design with the dimensions indicated in μm; the white dashed box indicates the intended position of the optical fiber after sensor assembly. (b) SEM of a longitudinal cross-section of the sensor element following TPP microfabrication. (c) SEM of a transverse cross section of the sensor element, excluding the distal section (region below the red dashed lines in Fig. 4(a)). The upper surface of the structure shown in this image corresponds to the cross-sectional plane indicated by the dashed red line in panel A, and the region labelled LS is the lensing segment, also shown in panels A and B. Complete washout of all excess unpolymerized photoresist can be seen in the inset [SM-OF: single-mode optical fiber, WH: washout hole, LS: lensing segment, AC: air cavity, DM: deformable diaphragm].

Importantly, to promote complete removal of unwanted photoresist following TPP illumination from the region designated as the air cavity, tapering washout holes/outlets (labelled WH) with bean-shaped cross-section were incorporated along the periphery of the lensing segment (away from the sensor interrogation beam path, Fig. 1(c)). The distal end edges of these washout holes were modified to a chamfered design to avoid build-up of uncured resist around sharp rims and recesses (a corresponding transverse cross-sectional CAD view is shown in Fig. 4(a)).

### B. Assessment of Fabrication Fidelity

Longitudinal and transverse cross-sections of the sensing element were fabricated with TPP using the same CAD design but bisected along its axes to expose the internal structure, to assess the internal fabrication fidelity of the sensor element. These cross-sectional sensor element structures were analyzed by optical microscopy, SEM and AFM. Optical microscopy and SEM of the fabricated microstructures (Fig. 4(b) and (c)), demonstrated excellent visual correspondence with the sensor element CAD cross-section depicted in Fig. 4(a).

Further, through TPP fabrication of the transverse cross-section, and photoresist development using our modified protocol and microstructure detachment (see Materials and Methods), complete removal of all unpolymerized photoresist material through the designated washout holes was verified (as shown in Fig. 4(c) inset). It was notable that presence of tapered washout holes with chamfered inner edges significantly improved the development process, compared to untapered, unchamfered cylindrical washout holes where significant unpolymerized photoresist material was observed. Moreover, surface roughness of 37.1 nm (root mean squared value) was measured by AFM on the proximal surface of the deformable diaphragm (detailed in the Materials and Methods section), while surface discontinuities on the curved lens surface are estimated to be <350 nm by virtue of the TPP lithography system resolution [37], [41]. The fabrication fidelity of the functional elements of the microfabricated sensing structure was therefore verified to correspond well to the CAD design with the surface roughness along the beam path being lower than half the interrogation wavelength (see Materials and Methods).

### C. Sensor Element Integration

**Fig. 5.**
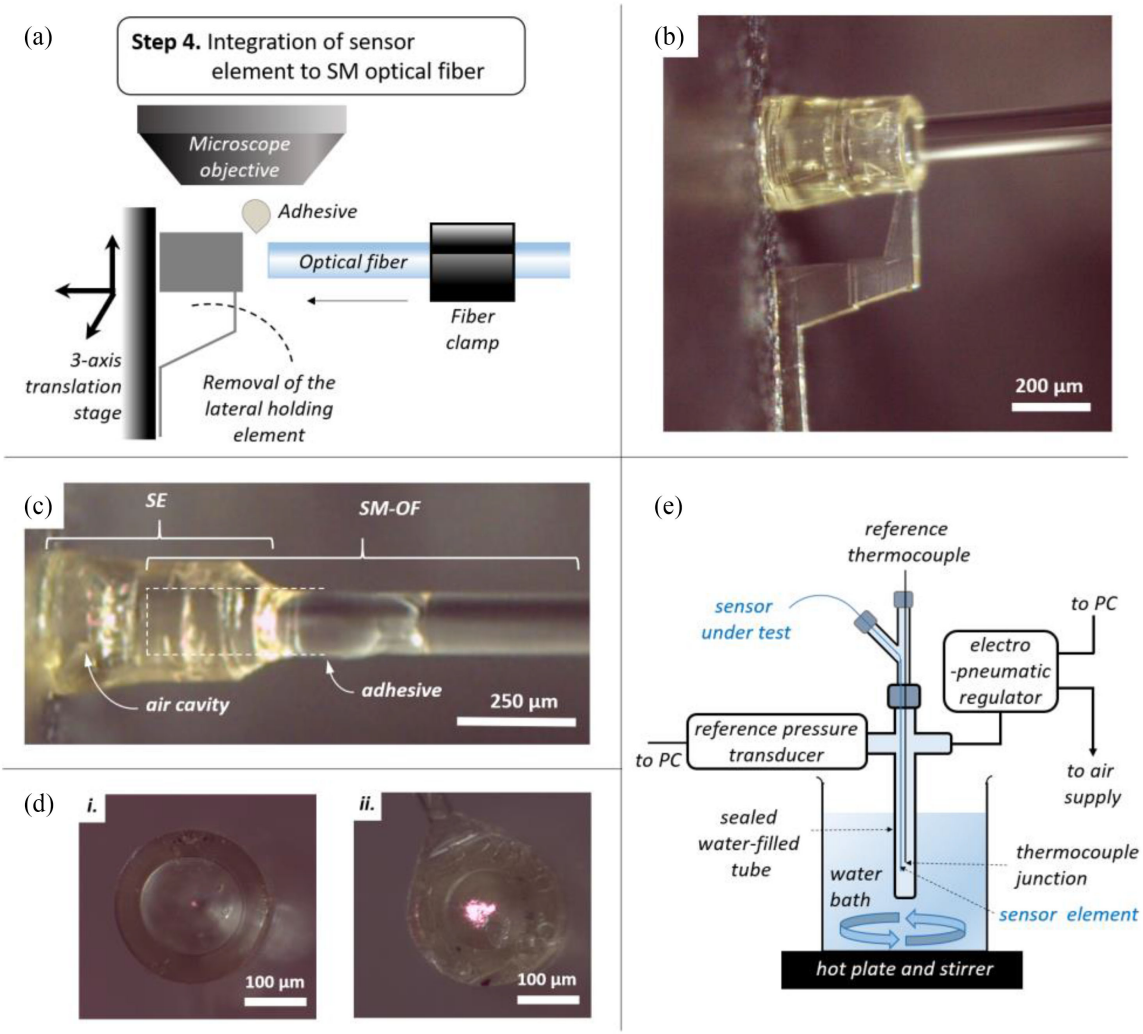
Sensor integration and characterization. (a) Schematic illustration of the sensor integration, following from the process flow of extrinsic sensor element microfabrication depicted in Fig. 3(a). (b) Microscope image of the optical fiber placement into the precision microfabricated extrinsic sensing element. (c) Microscope image of the integrated fiber-optic sensor. SE: sensing element, SM-OF: single-mode optical fiber. (d) Microscope images indicating the LCI interrogation beam spot in a fiber-optic sensor with *i*. S_lensed_ optimized lensed sensor design, and *ii*. S_ref_ reference sensor design with a flat central segment. These microscope images were obtained at SLD source intensities where sensor response signals could be discerned in each sensor design case. The higher apparent brightness in *ii*. compared to *i*. is due to the significantly higher source power required to obtain the adequate sensing signal in S_ref_ compared to S_lensed_. (e) Schematic illustration of the characterization setup to evaluate the pressure and temperature response of the fiber-optic sensors. This arrangement was used to simulate the pressure and temperature environment within the intravascular environment in order to characterize the constructed fiber-optic sensors.

The TPP-printed extrinsic sensor element was bonded to an SM optical fiber (SM800, Thorlabs, NJ, USA) under an optical microscope, as illustrated in Fig. 5(a). Following substrate detachment, the sensor element was temporarily affixed (held on by electrostatic force) to a vertical optical post connected to a 3-axis manual translation stage facing the distal end of the cleaved SM optical fiber (Fig. 5(b)). The relative position of the extrinsic sensor element to the optical fiber was controlled by manual manipulation of the translation stage. The position of the distal end of the optical fiber within the extrinsic sensor element can be monitored with the phase-resolved LCI system (see Materials and Methods). Following SM fiber positioning as per design (position indicated as the shaded white rectangle in Fig. 4(a)), a small amount of transparent cyanoacrylate adhesive (Loctite 4902, Henkel Adhesives, USA) was applied to the proximal end of the sensor element and cured at room temperature by leaving it undisturbed for at least 15 minutes. In addition to bonding the microstructure to the fiber, this process helps seal the air cavity of the sensor. Thereafter, the lateral holding element was readily snapped off with ultrafine tweezers to yield the integrated sensor. Following the integration step, the SM optical fiber and the extrinsic sensor element were found to be well aligned by visual inspection (Fig. 5(c)).

Furthermore, the lensing capability of this integrated sensor (S_lensed_) was evaluated against a comparable reference sensor design (S_ref_) where the polymer block segment had a flat surface instead of a curved lensing surface (described in Fig. 2). On inspection under a light microscope, the beam spot observed at the outer surface of the deformable diaphragm was found to diverge significantly more in S_ref_ compared to S_lensed._ (Fig. 5(d)). These experimental results are in agreement with the raytracing simulations corresponding to the optical designs for S_lensed_ and S_ref._ Moreover, a reduced illumination was required in the interrogation of S_lensed_ compared to S_ref_, while also showing an increase of ∼8.6 dB in the phase peak signal corresponding to cavity ***z*_2_** (the optical cavity formed by the confined air cavity segment bound on one side by the distal pressure-sensitive deformable diaphragm (PD), indicated in Fig. 1(b)). This confirmed the rationale for our micro-lensed fiber-optic sensor design optimization approach.

## IV. Sensor Response

The temperature and pressure-dependent sensor signals were obtained from the optical interrogation system (Fig. 1(d)), by phase-resolved low coherence interferometry (LCI). Details of the LCI system, pressure-temperature response characterization setup and evaluation of sensor signals ${\phi _1}$ and ${\phi _2}$ are given in the Materials and Methods section.

Sensor response was characterized in a simulated intravascular environment, in the form of a water-filled tube where the pressure and temperature could be controlled within the physiological range. The characterization setup implemented in this study is described in the Materials and Methods section, and schematically illustrated in Fig. 5(e). The optical cavity formed by the lensing segment of optical path length ***z*_1_** was found to be non-responsive to changes in pressure. This demonstrated the strong adherence of the microfabricated structure to its SM optical fiber, shielding the lensing segment from external hydrodynamic pressure variations. Concurrently, the distal deformable diaphragm was found to be responsive to these changes in external pressure.

**Fig. 6.**
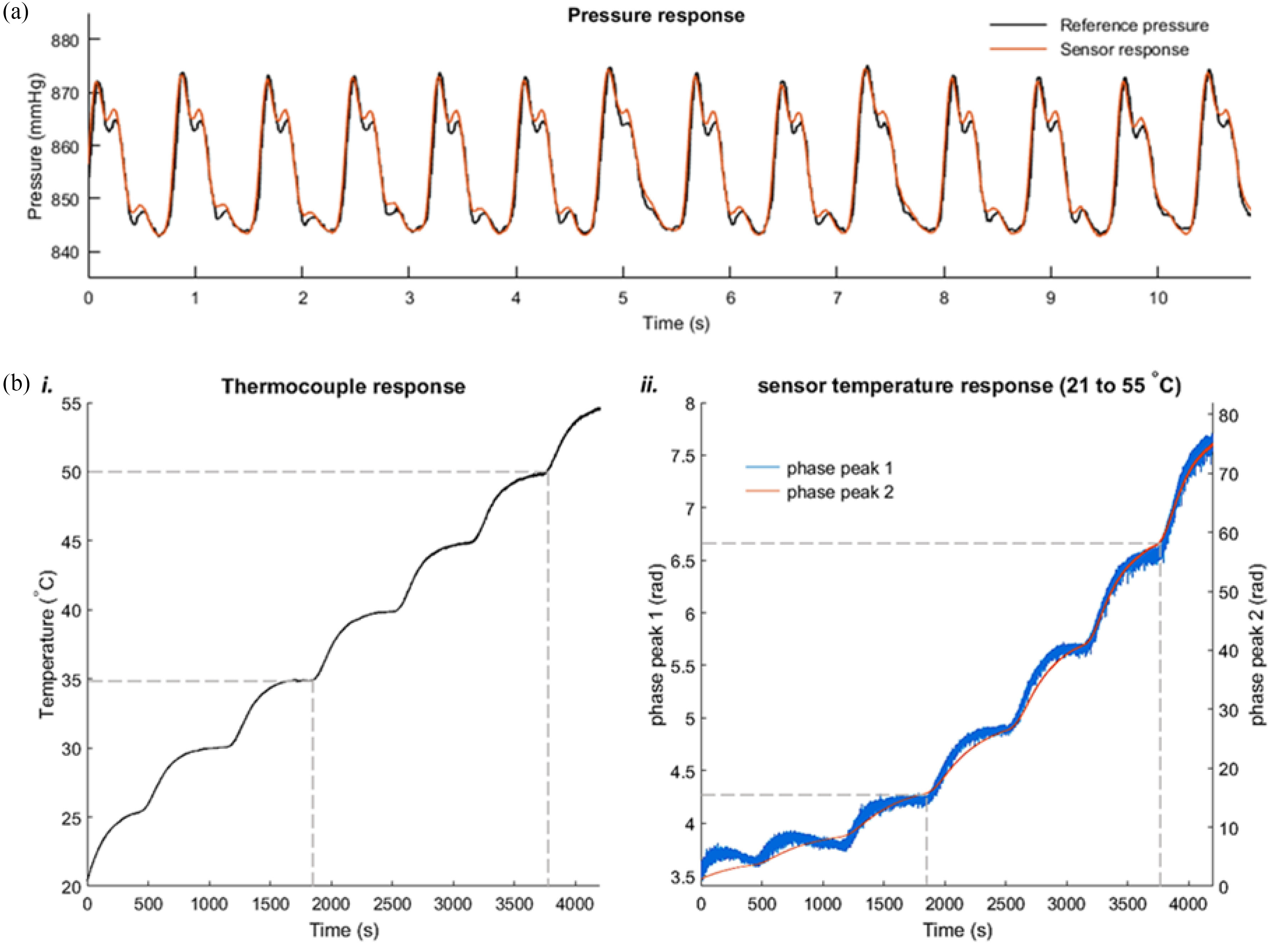
(a) Pressure response of the TPP printed sensor element under simulated arterial pulse response (72 pulses-per-minute). The pressure values are absolute, with an atmospheric pressure of 760 mmHg. (b) Temperature response of the TPP-printed sensor element under constant atmospheric pressure. i. Reference thermocouple response; ii. Sensor signals corresponding to the distal end of the lensing segment, and the proximal surface of the distal deformable diaphragm (φ1 and φ2 corresponding to optical path lengths z1 and z2, as described in the Materials and Methods section); the region bound by the dotted lines indicates temperature response in the range 34 °C to 50 °C.

The pressure response of this sensor was tested and found to approximate linear behavior in comparison to the bulk pressure reference sensor in the characterization setup. Evaluating the response of these microfabricated sensors in the range of 760 mmHg to 1060 mmHg (absolute pressures, with an atmospheric pressure of 760 mmHg; intravascular pressure sensors are typically tested in the range of 0 to 300 mmHg above atmospheric pressure), the pressure sensitivity of cavity ***z*_2_** was found to be 0.019 rad/mmHg with a noise floor of 0.042 rad, resulting in a pressure resolution of 0.11 mmHg. Moreover, measurements with a simulated arterial pulse pressure waveform at 72 pulses per minute were obtained and calibrated as per the above mentioned linear fit, plotted in Fig. 6(a). It can be observed that the pressure signal of cavity ***z*_2_** (i.e., distal deformable diaphragm) agrees well with the reference pressure sensor. Over longer duration measurements exceeding 30 minutes however, signal drift was found to be present (similar to other polymer-based interferometric sensors in literature) [23], [42], [43].

Response of the sensor to temperature changes was evaluated by applying controlled thermal gradients to the sensor by implementing a programmable temperature feedback in the outer water bath (Fig. 5(e)). Care was taken to place the reference thermocouple as close as possible to the sensor-under-test (<1 mm apart by visual inspection) to minimize the effects of local thermal gradients during testing/calibration measurements. Temperature changes in the range spanning 21 °C to 55 °C were induced (reference thermocouple signal and sensor response signals φ_1_ and φ_2_ are plotted in Fig. 6(b)). Within this range, the response was found to fit a quadratic relationship with the reference thermocouple. On closer inspection, the sensor signals’ response to temperature in the range of 35 °C to 50 °C (marked by the grey dotted lines in Fig. 6(b) ***i***), was found to approximate linear behavior with an R^2^ value of 0.981. In this temperature range, the sensitivity of φ_1_ (corresponding to cavity ***z*_1_*)*** was found to be 0.052 rad/ °C, with a noise floor value of 18.9 mrad (standard deviation of signal over a measurement duration of 10s). The temperature resolution was found to be 0.036 °C in this case.

The temperature response in these optical probes depend upon the thermal expansion and thermo-optic coefficient (change in refractive index with response to temperature) of the extrinsic sensing element material, in this case the proprietary IP-S photoresist. Non-linear temperature response beyond 50 °C could be attributed to the thermal expansion of the polymeric sensor element in 3D not being completely anisotropic along the optical axis in our sensor design. These dynamics could be varied and studied in detail by implementing modified TPP-printed multi-cavity sensor designs ensuring anisotropic thermal expansion in a large temperature range, or by using alternate materials, such as glass or metallic composites. High resolution TPP fabrication of these materials was recently reported and could be modified for implementation to fiber-optic probes [44], [45]. Moreover, it is expected that the pressure response of the sensor could be tuned through sensor design modifications, particularly the distal deformable diaphragm dimensions.

Further, these fiber-optic sensors were found to be robust following multiple measurement cycles by varying the pressure and temperature in the physiological range (stated in the Materials and Methods section). The bonding of the extrinsic sensing element to the optical fiber (as viewed under an optical microscope) and the sensor response (interrogated using the LCI system) were found to remain undamaged even after immersion in a water bath for a duration of 14 days. Additionally, the sensor signals were observed to be intact following mechanical impacts against the inner walls of the sealed water-filled polycarbonate tubes in the characterization setup (Fig. 5(e)), attesting to the sensor's structural ruggedness. Furthermore, for *in vivo* use, these sensors would be built into a catheter, guidewire or neede, lending additional mechanical robustness to the sensors.

The sensor response, robustness, geometry and chemical inertness in an aqueous environment is promising for its use as a pressure-temperature probe within human vasculature. Although blood differs from water in its viscosity, surface tension, refractive index and chemical composition, the authors are not aware of any particular concerns with the use of this sensor in blood. Though use of inert materials for the sensor construction, i.e., inert polymer, bonding adhesive and glass optical fiber, chemical interactions can be avoided. Further since the sensor interrogation is based on reflectance of the distal deformable diaphragm, the effects of optically scattering components of blood on its outer surface are expected to be negligible, given the low refractive index difference between red blood cells and blood plasma compared to the refractive index difference between air on the inner surface of the sensor and the distal polymer diaphragm [46].

## V. Summary and Future Perspectives

This work demonstrates the construction of a fiber-optic interferometric probe made as a monolithic extrinsic FP structure consisting of a confined gas-cavity and lensing segments with freeform geometry. The confined gas (air) cavity was constructed using a novel method that involved high resolution TPP 3D printing, with a freeform design suited to simple and irreversible bonding to an SM optical fiber for measurements in a remote fluidic environment. The optical design of the monolithic multi-cavity extrinsic sensing structure was optimized through raytracing simulations to minimize beam divergence within its optical cavities to improve sensor SNR for efficient performance. Importantly, the fiber-optic integration was performed using non-hygroscopic transparent cyanoacrylate adhesive to enable irreversible bonding of the sensing element to the optical fiber to enable prolonged operation within an aqueous environment, for instance the intravascular space. The technique described here lends well to high resolution micro-sensor prototyping with superior structural fidelity and design flexibility. Moreover, interrogating these low-finesse cavity fiber-optic sensors with phase-resolved LCI in a simulated intravascular environment yielded pressure and temperature sensitivities well within the range required for intravascular sensing catheters and intracranial pressure measurements as per medical device standardization requirements (FDA title-21B §870.1200 and §882.1620 respectively) [28]. The sensor constructed in this work is capable of measuring both parameters simultaneously with minimum detectable values of pressure and temperature being 0.11 mmHg and 0.036 °C respectively, albeit with some drift in long-duration measurements.

While the proof-of-concept design implemented in this paper comprised within a single microstructure - a microlens, air-cavity and a robust microscale polymeric diaphragm supported only at its edges, there is scope for inclusion of additional cavity elements with tunable mechanical and optical functionalities. Instead of forming an air cavity, additional materials (high-index resists, luminescent fluids, optical scatterers, or alternative gaseous substances) could be introduced into the TPP structure by micro-pipetting into the air cavity spaces of such fiber-optic probes for requisite optical, bio-chemical and mechanical sensing operations. Moreover, beyond the use of photoresists, this technique could be extended to multiphoton 3D printed fiber-optic extrinsic probes where the microscale extrinsic sensing structures are formed using alternate materials such glass or metals to lower sensor drift, and/or for operation in other measurement ranges.

In addition to extending this sensor construction paradigm to myriad materials for precision TPP-based microfabrication, design modifications for alternative sensing applications could be explored. For instance, a single air-filled optical cavity design could be adapted to form miniature high-finesse cavities for fiber-optic ultrasound sensing by metallizing the distal end of the optical fiber and proximal end of the deformable diaphragm to enhance cavity finesse. Along with the interferometric measurement of physiological pressure and temperature, such sensors could be modified for multiplexed sensing and biomolecule detection by including biochemically functionalized optically responsive external surfaces of the distal deformable diaphragms. The optical design may also be modified to deliver focused optical energy for tissue ablation, light-guides for mm-scale endoscopic cameras and/or diffractive optical elements integrated to optical fibers for measurements in opto-genetics. Moreover, use of recently developed photoresist material with long-term biocompatibility (for instance IP-Visio) to form the probe microstructure could enable development of minimally invasive implantable devices.

Combined with the ongoing innovation in parallelized femtosecond laser-based material processing and adaptive optics, these developments could enable large-scale production of precision microfabricated fiber-optic probes for use in a variety of areas within and beyond biomedical sensing. Therefore, this work presents significant inroads in the field of fiber-optic sensors in general, and MIS probes in particular.

## VI. Materials and Methods

### A. LCI Setup

Sensor interrogation was performed through phase-resolved LCI with the sensing fiber connected in a Michelson interferometer configuration (Fig. 1(d)), based on the system described by Coote *et al.* [19] Briefly, broadband light from a superluminescent diode (SLD) light source with full-width-at-half-maximum (FWHM) bandwidth of 64 nm centered at 820 nm and output power of 15mW (BLM-S-280-B-I-10; Superlum, Ireland) was guided to the fiber-optic sensor via a 50:50 fiber coupler (TW850R5A2,Thorlabs, NJ, USA). An in-line attenuator (VOA-850-APC, Thorlabs) was placed between the source and the coupler to allow adjustment of the incident power. The back-reflected light from the sensor was directed to a spectrometer (Maya 2000Pro, Ocean Optics, FL, USA) via a fiber coupler. A custom LabVIEW (National Instruments, Newbury, U.K.) program was used to acquire and process the raw spectra, at a sampling rate of up to 250 Hz.

### B. Phase-Resolved LCI

The LCI sensing mechanism can be described by reference to the two-cavity design of the extrinsic sensing element, with optical cavity lengths indicated as ***z*_1_** and ***z*_2_** in Fig. 1(b). Variation in local pressure and temperature induce changes in the lengths of these cavities. Increases in pressure in the surrounding medium cause the distal deformable diaphragm (PD) to deform, causing a change in the cavity length ***z*_2_** by ***Δz*_2_**. A temperature increase causes the photoresist material to expand, modifying the cavity length ***z*_1_** by ***Δz*_1_**. The distal deformable diaphragm also undergoes thermal expansion contributing to ***Δz*_2_**, depending on the design of the sensor element. These changes in optical path length can be monitored as variations in the complex argument of the inverse Fourier-transformed spectrum at its maxima, as detailed in our previous work [19], [38].

Briefly, an inverse Fourier transform (IFT) is applied to the intensity spectrum obtained from the spectrometer. The maxima of the complex magnitude of the IFT can be shown to be proportional to the baseline optical cavity lengths, ***z_1_*** and ***z_2_***, and small temperature and pressure-induced variations in these optical cavity lengths, ***Δz*_1_** and ***Δz*_2_**, can be obtained from the complex argument of the IFT at these maxima, according to Choma *et al.*, [47]

\begin{equation*}
{\phi _j}\left({t - {t_0}} \right){\rm{\ }} = \arg \left\{ {{\Im ^{ - 1}}\left[ {I\left(k \right)} \right]\left({ + {z_j}} \right)} \right\}{\rm{\ }} = \frac{{2\pi }}{{{\lambda _c}}}\ \Delta {z_j}\left({t - {t_0}} \right)
\end{equation*}

Where *I*(*k*) is the intensity spectrum as a function of wavenumber **k**, ***z_j_*** is the baseline optical cavity length, which includes the geometric length and refractive index, *j* is an integer equal to 1 or 2 signifying each of the two optical cavities, λ_c_ is the central wavelength of the SLD, and ***t*** is time, referenced to a start time ***t*_0_**_._ The complex argument signal, φ_j_ forms the output of the sensor. In this work, two such complex argument signals φ_1_ and φ_2_, corresponding to the optical cavity lengths ***z*_1_** and ***z*_2_** were analyzed. These signals were calibrated against reference pressure and temperature sensors, to allow measurement of absolute changes in pressure and temperature using this fiber optic probe.

### C. Ray-Tracing Simulation

Ray tracing for the optical system representing the sensor design was performed on OpticStudio 15.6 software (Zemax LLC, Washington, USA) using the Physical Optics propagation method in the Sequential ray tracing mode. The basic design paradigm used for these structures is indicated in Fig. 1(b). After emanating from the core of a SM optical fiber cleaved at normal incidence, the interrogation beam passes through the photoresist material (LS), then through the air cavity (AC), before being back-reflected along the same path to the optical fiber core. The SM optical fiber core was simulated as a 5.6 μm diameter Gaussian light source of wavelength 820 nm (the central wavelength of the SLD source in our LCI interrogation system), and a numerical aperture of 1.4 obtained from the technical specifications of the optical fiber used (SM800, Thorlabs). At this wavelength, the refractive index of the constituent photoresist material of our sensor (IP-S) was taken as 1.49 from the measurements conducted by T. Gissibl, *et al.*, [48] while the deformable diaphragm was modelled as a partial reflector (reflectance of 50%) of thickness 3 μm.

The surface curvatures and dimensions of the various cavity elements were optimized, while also considering possible positioning errors during sensor integration by relative light source decentering (optical fiber core misalignment) by ±1 μm in the optical system Two cases of decentered source are depicted as the red and green paths and the case of centered source as the blue path in Fig. 1(c). Moreover, in this illustration of the ray trace optimization, the forward and back-reflected beam trajectories are shown separately with the trajectory directions indicated by arrows (instead of geometrically superimposed forward and back-reflected trajectories after beam reflection at the distal air-diaphragm interface).

### D. TPP Microfabrication and Substrate Detachment

The complete sensor element, including the lateral holding element, was designed using computer aided design (CAD) software (Autodesk Inventor, Autodesk Inc., San Rafael, CA, USA). This 3D CAD design for the sensor element was converted from Standard Tessellation Language (STL) format to the native General Writing Language (GWL) data format of the TPP system using DeScribe software (Nanoscribe GmbH). Subsequently, the GWL file was loaded into the system's control software NanoWrite (Nanoscribe GmbH) for print execution.

The extrinsic sensor element was fabricated on a glass substrate coated with Indium Tin Oxide (ITO), using a commercial TPP system (Photonic Professional-GT, Nanoscribe GmbH) and its 25x objective. During TPP printing, polymerization inside a droplet of liquid UV curable resin (IP-S, Nanoscribe GmbH) was induced at the focus of a 780 nm femtosecond laser beam by two-photon absorption. The beam could be rapidly steered laterally (X-Y) within a hexagonal block (350 × 350 × 200 μm). For fabricating structures larger than the predefined microscale volume, printing was performed across multiple parallelepiped blocks angled along the z-axis at 10°. The complete structure was realized by stitching together individual blocks such that no slice lines fell along the path of the sensor interrogation beam path designated in the ray trace, with some additional allowance along the periphery (cylindrical region coaxial with the sensor element, of 50 μm radius). The average fabrication time for each sensor element was 54 minutes (19 minutes when excluding the lateral holding element section) using the above mentioned commercial TPP system and print settings.

Following TPP printing, the microstructures were developed by immersing in propylene-glycol monomethyl-ether-acetate (PGMEA) for 180 minutes to ensure complete washout of uncured resist from the complete structure, and subsequent rinsing in isopropyl-alcohol (IPA) for 15 minutes was performed to ensure removal of residual developer. The 3D printed structures were lifted-off the ITO-glass substrate with extra-fine tip, acid- resistant tweezers. These steps are represented schematically in Fig. 3(a).

### E. Surface Roughness Measurement

The surface roughness of the proximal surface of the diaphragm was characterized using an AFM (Veeco Dimension 3100) in a transverse cross-section of a test sample consisting of only 45 μm of the distal-most section of the sensing element, to allow the AFM tip to access the measurement surface. The average surface roughness values were obtained by evaluating the AFM data on NanoScope Analysis software package in the tapping mode over an area of 5 μm square.

### F. Physical Simulation of Intravascular Pressure and Temperature Variation

The pressure and temperature responses of the sensor were measured with a pressure-sealed water chamber. Pressure within this chamber was varied with an electro-pneumatic regulator (ITV-0010-3BS, SMC, Japan) and monitored with a custom configured pressure transducer (MMA030-USBHB3MC0T8A6CE, Omega Engineering, CT, USA). Pulsed pressure variations with different step sizes and duty cycles were induced within this chamber across the absolute range of 760 mmHg (atmospheric) to 1060 mmHg.

For temperature sensitivity measurements, the chamber was placed in a programmable water bath with its temperature varied from 21 to 55 °C. The pressure within the sealed water chamber was kept constant during temperature sensitivity measurements. The reference temperature sensing thermocouple (409-4908, RS Components, U.K.), was positioned adjacent to the precision 3D printed fiber-optic sensor. Interferometric phase changes were calibrated to values from the corresponding reference sensor with a linear transformation
